# Rolling Bearing Fault Diagnosis Based on an Improved HTT Transform

**DOI:** 10.3390/s18041203

**Published:** 2018-04-14

**Authors:** Bin Pang, Guiji Tang, Tian Tian, Chong Zhou

**Affiliations:** School of Energy, Power and Mechanical Engineering, North China Electric Power University, Baoding 071000, China; tanggjlk@ncepu.edu.cn (G.T.); nceputt@126.com (T.T.); zhouchong010@126.com (C.Z.)

**Keywords:** improved Hilbert TT transform, Hilbert TT transform, principal component analysis, rolling bearing, fault diagnosis

## Abstract

When rolling bearing failure occurs, vibration signals generally contain different signal components, such as impulsive fault feature signals, background noise and harmonic interference signals. One of the most challenging aspects of rolling bearing fault diagnosis is how to inhibit noise and harmonic interference signals, while enhancing impulsive fault feature signals. This paper presents a novel bearing fault diagnosis method, namely an improved Hilbert time–time (IHTT) transform, by combining a Hilbert time–time (HTT) transform with principal component analysis (PCA). Firstly, the HTT transform was performed on vibration signals to derive a HTT transform matrix. Then, PCA was employed to de-noise the HTT transform matrix in order to improve the robustness of the HTT transform. Finally, the diagonal time series of the de-noised HTT transform matrix was extracted as the enhanced impulsive fault feature signal and the contained fault characteristic information was identified through further analyses of amplitude and envelope spectrums. Both simulated and experimental analyses validated the superiority of the presented method for detecting bearing failures.

## 1. Introduction

As a key component of rotating machinery, rolling bearings are responsible for ensuring the rotation accuracy of shafts and providing stable support for machines, so their running state is directly related to the performance of the entire system [1,2,3]. Severe running conditions cause bearings to be prone to various local damage faults, such as pitting, wear, and cracks, which can bring a series of adverse effects. Monitoring bearing conditions and detecting potential failures is important to ensure production safety and avoid unnecessary economic losses.

In past decades, various diagnostic methods, such as oil sample detection [4], acoustic emission [5,6,7,8], and vibration analysis [9,10,11], have been employed to diagnose rolling bearing faults, and the vibration signal based approach is seen as the most effective and convenient method. Numerous signal analysis methods have been researched to process bearing fault vibration signals for fault feature extraction. In general, these methods can be classified into three categories: time domain, frequency domain and time–frequency domain [12]. In time domain analysis, time domain statistic indexes, such as root mean square, kurtosis, and skewness [13], are utilized to evaluate the health conditions of rolling bearings. However, time domain statistics indexes are usually insensitive to incipient faults and are unable to identify specific fault types [14]. When local damage faults appear in a bearing, its vibration signal exhibits periodic impacts and frequency domain approaches are commonly used to discriminate the characteristic frequencies of these impacts. Conventional frequency domain analysis techniques include fast Fourier transform (FFT) spectrum [15], Hilbert transform (HT) envelope spectrum [16], bisepctrum [17], Teager energy spectrum [18], and spectral kurtosis (SK) [19]. One challenge of frequency domain analysis techniques is that fault feature signals are very weak relative to background noise and other interferences in the early damage stage; thus, conventional spectrum methods will lose efficacy in their fault diagnosis of rolling bearings. Therefore, frequency domain analysis methods need to be combined with fault feature enhancement methods, such as signal de-noising methods [20,21], deconvolution methods [22,23,24], and pre-whiten methods [25,26], to complete early fault diagnosis in many cases. Time–frequency domain approaches, such as short-time Fourier transform [27], wavelet transform [28], and Hilbert Huang transform [29], have been shown to be more suitable for extracting non-stationary and time-varying fault feature information [30]. Unfortunately, traditional time–frequency methods are also easily affected by noise and have some limitations [31]. It is critical to take measures to enhance fault feature signals.

The S transform, developed from a wavelet transform, is a superior time–frequency technique that has high time frequency resolution and is more adaptive and robust than a wavelet transform [32]. A novel signal processing technique that originated form S transform, called time–time (TT) transform, has been proposed as a time–time domain analysis method that can provide a better view of the time-local properties of signals [33,34] and it has been shown to be useful for the applications of power system fault detection [35] and power event identification [36]. Based on the TT transform, a Hilbert TT (HTT) transform was proposed by Xianfeng Fang and Ming J Zuo [37] to extract the modulating signal for gearbox fault detection by combining a HT with TT transform. This new method was introduced to reveal the impulses buried in the vibration signals of faulty bearings. To eliminate the effect of noise on the HTT transform, we present an improved HTT transform (IHTT) method by introducing principal component analysis (PCA), de-noising the HTT transform and investigating its effectiveness by simulation and experimental verification.

The contents of the following sections are as follows. Section 2 introduces the basic theories of HTT transforms and PCA. Section 3 gives the specific steps of the IHTT transform. Simulated and experimental verifications are conducted in Section 4 and Section 5 and conclusions are summarized in Section 6.

## 2. Basic Theories

### 2.1. Hilbert Time–Time Transform

The HTT transform comes from the combination of HT and TT transforms. The original signal is firstly analyzed by HT to get the instantaneous envelope signal and the instantaneous envelope signal is further analyzed by a TT transform to get the HTT transform results [37]. The following is a detailed introduction to the two main steps of HTT transforms. 

The HT of a given signal *x*(*t*) is expressed as [38]:

(1) $$y(t)=\frac{1}{\pi }\int_{-\infty }^{\infty }\frac{x(u)}{t-u}du$$

The analytic signal *Z*(*t*) is then obtained through Equation (2):

(2) $$Z(t)=x(t)+iy(t)=a(t)e^{i\varphi (t)},$$

in which *a*(*t*) denotes the instantaneous envelope, and *φ*(*t*) is the phase function. Equation (3) and Equation (4) are the expressions of *a*(*t*) and *φ*(*t*), respectively:

(3) $$a(t)=\sqrt{x^{2}(t)+y^{2}(t)},$$

(4) $$\varphi (t)=\arctan\frac{y(t)}{x(t)}.$$

The instantaneous envelope signal is used as the input signal to perform the TT transform. The TT transform comes from the inverse Fourier transform of the S transform. Equation (5) shows the S transform of the instantaneous envelope signal:

(5) $$S(f,\tau )=\int_{-\infty }^{+\infty }a(t)\frac{\left|f\right|}{\sqrt{2\pi }}e^{-\frac{f^{2}{\left(\tau -t\right)}^{2}}{2}}e^{-i2\pi ft}dt,$$

where, *f* and *τ* denote frequency and time, respectively.

Then, we get the TT transform of *a*(*t*):

(6) $$TT(t,\tau )=\int_{-\infty }^{+\infty }S(f,\tau )e^{i2\pi ft}df.$$

As reported in [33], the time–time map of *TT*(*t*,*τ*), which is also called the TT transform spectrum, obtained by Equation (6) gives the time–time domain representation of the original signal. The time intervals of *a*(*t*) can be identified in this map. The method has an important feature that elements with high frequencies possess high amplitudes near *t* = *τ* in the TT transform spectrum. This feature can be used to derive the periodic impact signal from the low-frequency harmonic signals by selecting the diagonal data of the matrix *TT*(*t*,*τ*) as the fault feature signal.

### 2.2. Principal Component Analysis for Matrix De-Noising

Principal component analysis has shown its effectiveness in extracting main information from high-dimension data [39,40]. It is used for matrix de-noising in this paper.

Given an original matrix ***X***∈***R****^n^*^×*m*^:

(7) $$X=\left[\begin{array}{cccc} x_{11} & x_{12} & \cdots & x_{1m}\\ x_{21} & x_{22} & \cdots & x_{2m}\\ \vdots & \vdots & \ddots & \vdots \\ x_{n1} & x_{n2} & \cdots & x_{nm} \end{array}\right].$$

***X*** can also be written as ***X***=[***x***_1_, ***x***_2_,…,***x****_m_*], and ***x****_i_*=(*x*_1*i*_, *x*_2*i*_,…,*x_ni_*)^T^. 

Let ***C*** be the covariance matrix of ***X***, and *λ_i_* (*i*=1,2,…,*m*) ordered from big to small are the eigenvalues of ***C***. ***α****_i_*=(*α*_1*i*_, *α*_2*i*_,…,*α_mi_*)^T^ represents the eigenvector corresponding to the *i-*th eigenvalue *λ_i_*. 

The *m* projection vectors can be derived from Equation (8):

(8) $$\begin{array}{c} y_{1}=\alpha_{11}x_{1}+\alpha_{21}x_{2}+\cdots +\alpha_{m1}x_{m}\\ y_{2}=\alpha_{12}x_{1}+\alpha_{22}x_{2}+\cdots +\alpha_{m2}x_{m}\\ \vdots \\ y_{m}=\alpha_{1m}x_{1}+\alpha_{2m}x_{2}+\cdots +\alpha_{mm}x_{m} \end{array}.$$

Each projection vector ***y****_i_* in Equation (8) corresponds to the *i-*th eigenvalue *λ_i_* with the expression:

(9) $$y_{i}=X\alpha_{i}(i=1,2,\cdots ,m).$$

Previous studies show that ***α****_i_* has the property shown in Equation (10):

(10) $$\sum_{i=1}^{m}\alpha_{i}\alpha_{i}{}^{T}=I.$$

According to Equation (9), Equation (11) can be derived:

(11) $$\sum_{i=1}^{m}y_{i}\alpha_{i}{}^{T}=\sum_{i=1}^{m}X\alpha_{i}\alpha_{i}{}^{T}.$$

Substituting Equation (10) into Equation (11), we get:

(12) $$X=\sum_{i=1}^{m}y_{i}\alpha_{i}{}^{T}.$$

Among the *m* projection vectors, some projection vectors can be seen as the principal components. Then, we can select the former *l*(*l* ≤ *m*) principal components to reconstruct the estimation matrix shown below and realize the purification of the original matrix.

(13) $$\hat{X}=\sum_{i=1}^{l}y_{i}\alpha_{i}{}^{T}.$$

The common ways to choose the effective principal components and the corresponding eigenvectors in the related reports are based on the characteristics of the eigenvalues. In this paper, the difference spectrum of eigenvalues, which is similar to the concept of the difference spectrum of singular values in [41], was used to adaptively find the boundary point to separate the effective eigenvalues from the invalid eigenvalues. First, the forward difference of eigenvalues is shown below:

(14) $$b_{i}=\lambda_{i}-\lambda_{i+1},\;i=1,2,\cdots ,m-1.$$

The difference spectrum of eigenvalues is defined as the sequence ***B***= (*b*_1_, *b*_2_,…,*b_m_*_-1_). If the value of the difference of two adjacent eigenvalues is close to zero, the remaining eigenvalues tend to be stationary and they are thought to contribute to the noise components. There are some peaks in the difference spectrum of the eigenvalues, principal components and eigenvectors corresponding to the eigenvalues before the order *k* (*b_k_* exists the last significant peak in the difference spectrum eigenvalues) are selected to reconstruct the de-noised matrix, as Equation (15) displays.

(15) $$Y=\sum_{i=1}^{k}y_{i}\alpha_{i}{}^{T}.$$

## 3. Improved Hilbert Time–time Transform

**Figure 1:**
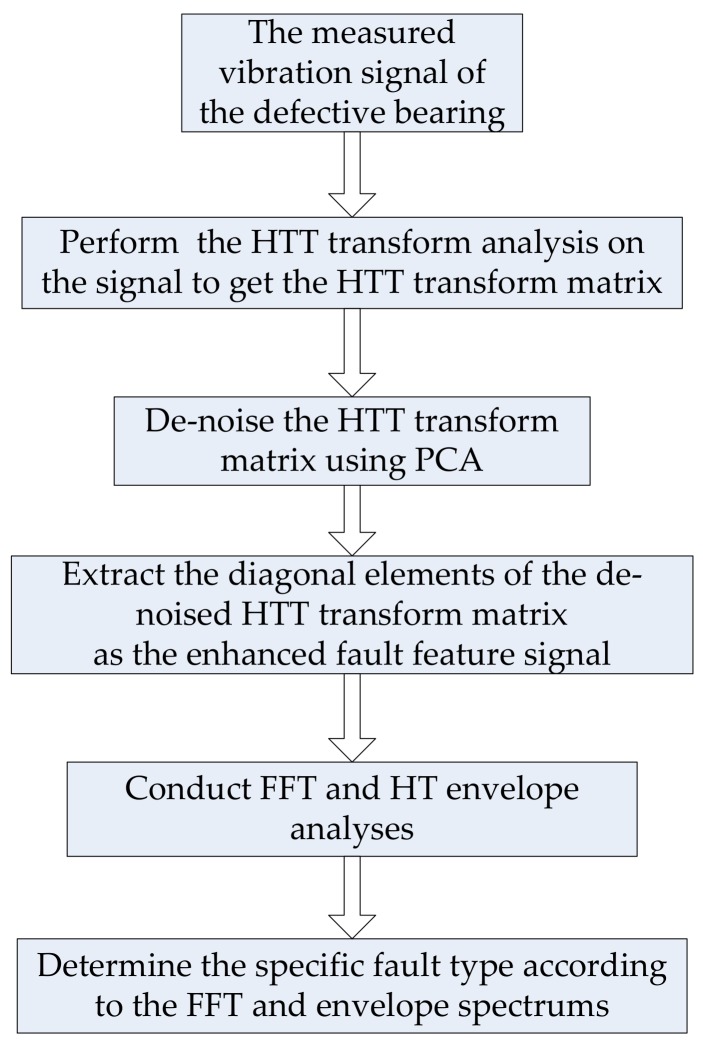
Block diagram of the improved Hilbert time–time (IHTT) transform for bearing fault diagnosis.

A two-dimensional time–time matrix, namely a HTT transform matrix, in this paper was expected to be achieved after applying the HTT transform to a time signal. When an original signal contains a certain amount of noise, the HTT transform matrix is redundant. The diagonal elements of the HTT transform matrix will also be affected, which will make it hard to identify the shock features. It is useful to take some measures to inhibit the influence of noise on the HTT transform. Therefore, the IHTT transform method is proposed, where PCA will be applied to de-noise the HTT transform matrix. The block diagram of the IHTT transform for bearing fault diagnosis is depicted in Figure 1, and the specific implementation steps are as follows:

1. Apply the HTT transform to the measured vibration signal to get the HTT transform matrix.
2. Employ PCA de-noising of the HTT transform matrix to get the de-noised HTT transform matrix.
3. Extract the diagonal elements of the de-noised HTT transform matrix to construct the enhanced fault feature signal.
4. Conduct FFT analysis and HT envelope analyses on the enhanced fault feature signal to get the FFT and envelope spectrums.
5. Determine the specific fault type of the bearing according to the FFT and envelope spectrums.

## 4. Simulation Analysis

A multi-component signal *x*(*t*), shown in Equation (16), was constructed for simulation verification.

(16) $$\{\begin{array}{c} x(t)=x_{1}(t)+x_{2}(t)+x_{3}(t)\\ x_{1}(t)=\sin(2\pi \;f_{1}t)\\ x_{2}(t)=0.5\exp(-800t_{1})\sin(2\pi \;f_{n}t+2/\pi ),t_{1}=\mathrm{mod}(t,1/f_{o})\prime \\ x_{3}(t)=0.1\;rand(1,N) \end{array}$$

where *x*_1_(*t*) is a sinusoidal signal with the frequency *f*_1_ = 30 Hz, employed to simulate harmonic interference. *x*_2_(*t*) is the simulated outer race (OR) fault signal of the rolling bearing constructed using the model in [42]. *x*_3_(*t*) represents the random noise that is generated with the matlab function randn(1, *N*), and *N*=1024 is the signal length. In the expression of *x*_2_(*t*), *f_n_*= 1024 Hz represents the inherent frequency and *f_o_*= 80 Hz denotes the characteristic frequency. The sampling frequency *f_s_*= 4096 Hz.

**Figure 2:**
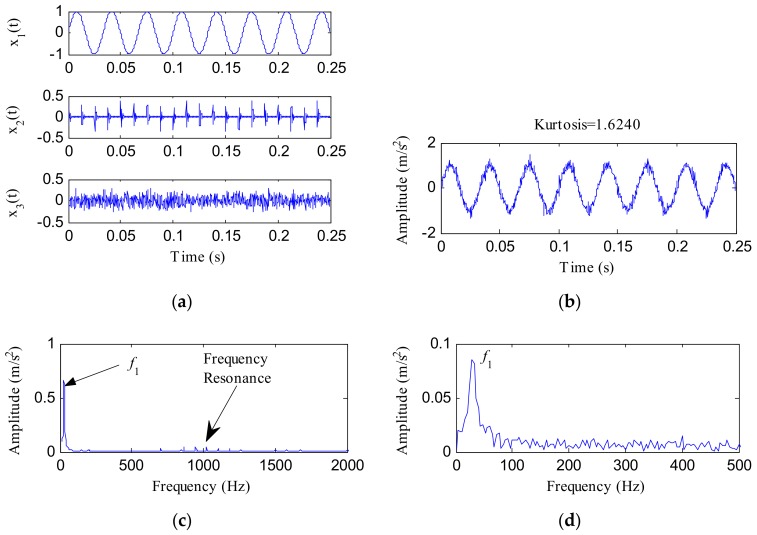
The simulated signal *x*(*t*): (**a**) composed components; (**b**) time waveform; (**c**) fast Fourier transform (FFT) spectrum; (**d**) envelope spectrum.

Figure 2 displays the composed components, time waveform, FFT spectrum and envelope spectrum of *x*(*t*). The kurtosis of *x*(*t*) was 1.6240. No periodic impacts were visible in the waveform of *x*(*t*). Additionally, both the FFT and envelope spectrums of *x*(*t*) were dominated by the frequency of the harmonic interference *x*_1_(*t*) and no fault features could be identified from them. 

**Figure 3:**
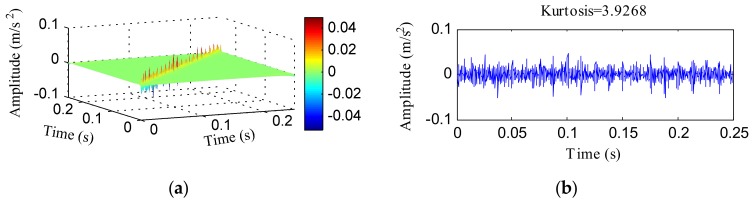

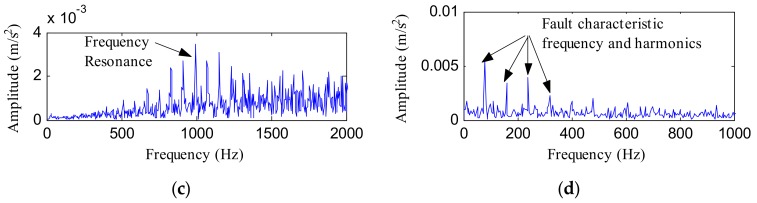
The analysis results of *x*(*t*) using the HTT transform: (**a**) the HTT transform spectrum; (**b**) the diagonal elements of (**a**); (**c**) the FFT spectrum of (**b**); (**d**) the envelope spectrum of (**b**).

The simulated signal *x*(*t*) was analyzed with an HTT transform directly and Figure 3 shows the results. Figure 3a reflects the HTT transform spectrum of *x*(*t*) and Figure 3b displays the data on its diagonal. The kurtosis of the signal shown in Figure 3b increased to 3.9268. The signal shown in Figure 3b exhibited some pulse impacts, but the pulse impacts were mixed with enough noise, which decreased the shock features. Figure 3c,d, respectively, shows the FFT and envelope results of the data in Figure 3b. Although frequency resonance was found, as shown in Figure 3c, the resonance band was narrow because some of the sidebands were overwhelmed by noise. From Figure 3d, some prominent peaks appeared at the frequencies of 80, 160, 240, and 320 Hz. The analysis results of the HTT transform demonstrate that an HTT transform can contribute to inhibiting harmonic interference, but that it is easily affected by noise.

**Figure 4:**
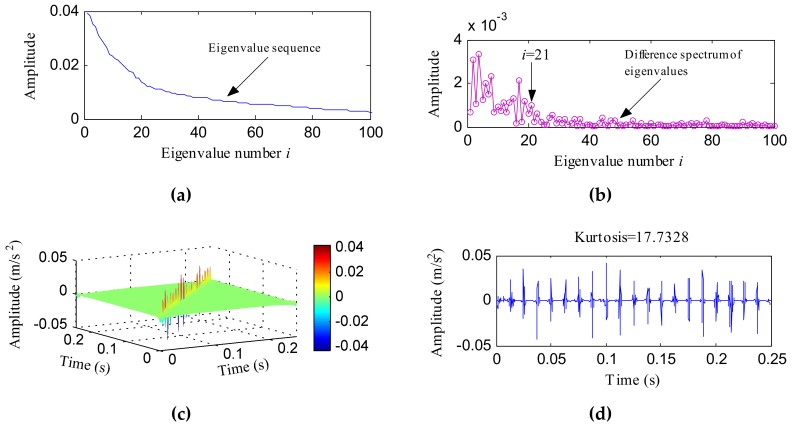

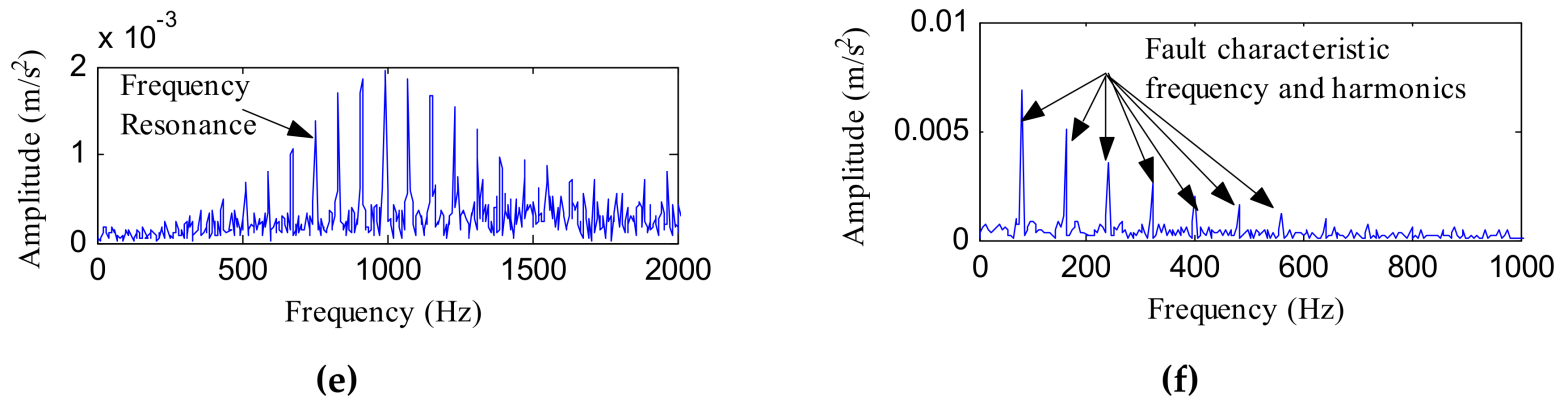
The improved Hilbert time–time (IHTT) transform process results of *x*(*t*): (**a**) the front 100 data of the eigenvalue sequence; (**b**) the front 100 data of the difference spectrum of eigenvalues; (**c**) the IHTT transform spectrum; (**d**) the diagonal elements of (**c**); (**e**) the FFT spectrum of (**d**); (**f**) the envelope spectrum of (**d**).

The simulated signal was then processed with an IHTT transform and Figure 4 shows the process. The HTT transform matrix was analyzed using the PCA method. The front 100 data of the eigenvalue sequence and the difference spectrum of eigenvalues are, respectively, plotted in Figure 4a,b. From Figure 4b, the last obvious peak appeared at the position of the 21st eigenvalue. Then, the front 21 principal components and the corresponding eigenvectors were used to reconstruct the de-noised matrix (as shown in Figure 4c), which was called the IHTT transform spectrum in this paper. Figure 4d shows the diagonal data with a kurtosis of 17.7328. The signal shown in Figure 4d had the same impulsive property as *x*_2_(*t*) and noise was completely suppressed. Meanwhile, the kurtosis value increased to 17.7328. Figure 4e,f displays the FFT and envelope results of the signal shown in Figure 4d, respectively. The band of frequency resonance reflected in Figure 4e was wider and clearer than that shown in Figure 3c. More harmonics of *f_o_* (80, 160, 240, 320, 400, 480, 560Hz) could be identified in Figure 4e than Figure 3d. The comparison analysis results demonstrate that the IHTT transform can overcome noise interference and improve the effects of the HTT transform.

**Figure 5:**
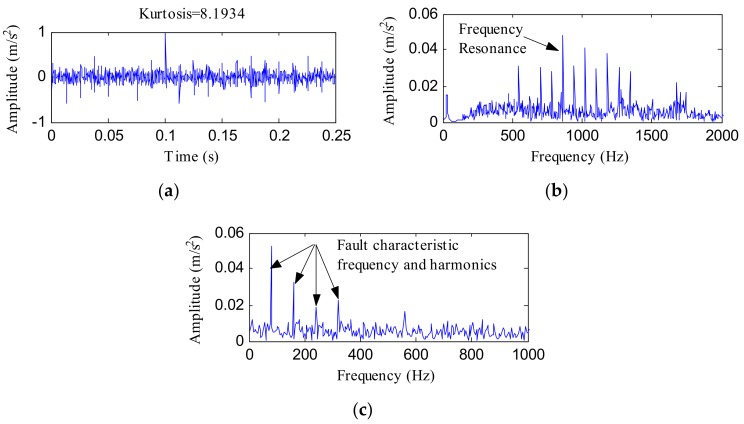
(**a**) Minimum entropy deconvolution (MED) filtered signal of *x*(*t*); (**b**) its FFT spectrum; (**c**) its envelope spectrum.

The minimum entropy deconvolution (MED) method [22] was employed to process the simulated signal for comparison. MED is a deconvolution method which enhances impact characteristics by eliminating the influence of transfer paths. Figure 5a displays the MED filtered signal. Its FFT spectrum and envelope spectrum are, respectively, displayed in Figure 5b,c. The kurtosis of the MED filtered signal climbed to 8.1934, which was higher than *x*(*t*) but lower than the enhanced fault feature signal obtained using the IHTT transform. The FFT spectrum and envelope spectrum contrast analysis results show that the proposed method is superior to MED in enhancing fault feature signals.

## 5. Applications

The proposed method was verified using two experimental samples: (a) An OR fault signal downloaded from the bearing data center of Case Western Reserve University (CWRU) [43]; (b) An inner race (IR) fault signal obtained by experimental simulation.

### 5.1. Case 1: Outer Race Fault Detection

**Figure 6:**
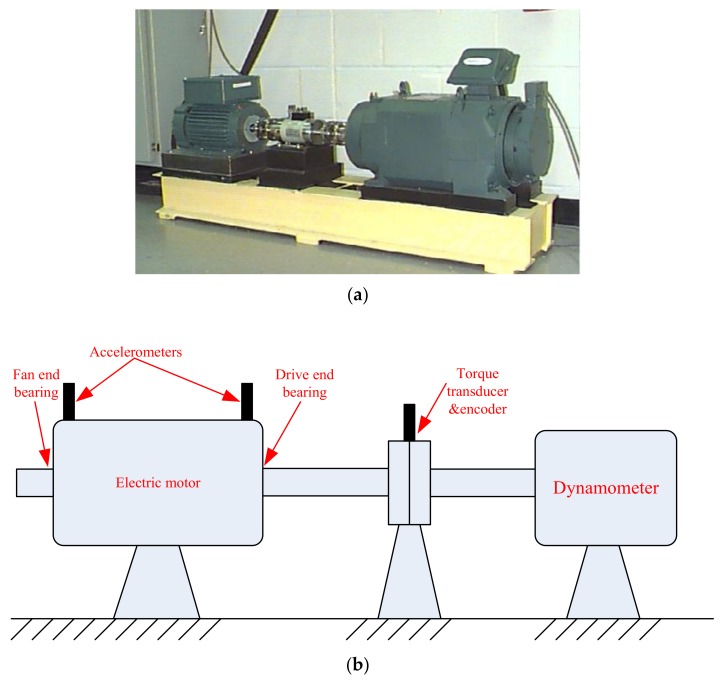
Test rig of case 1 and its structure diagram.

Figure 6 displays the test rig and its structure diagram. The test rig was composed of a motor (left), a torque transducer (center), and a dynamometer (right). The drive end bearing (SKF 6025 deep grove ball bearing) was studied. Table 1 lists the parameters of the bearing. The vibration signals were measured by accelerometers attached on the motor housing, as Figure 6 shows, and collected with a sampling rate of 12000 Hz. An OR fault sample with the defect size of 0.1778 mm was selected for analysis. The selected data were collected at the speed of 1750 rpm. The rotating frequency (*f_r_*) and the OR fault characteristic frequency (*f_o_*) were estimated as 29.17Hz and 104.5Hz, respectively.

**Figure 7:**
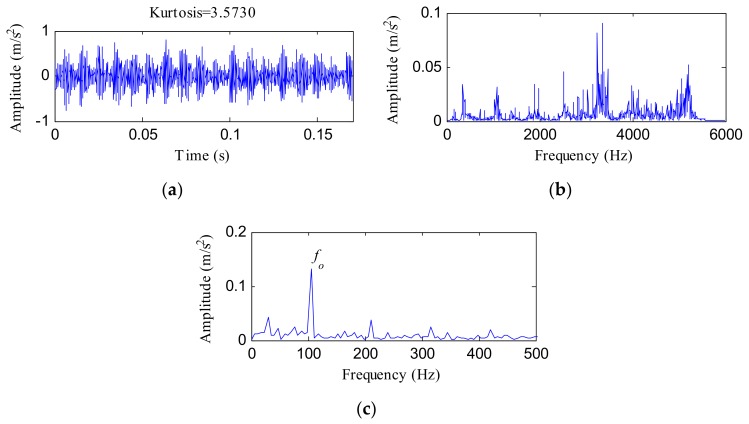
The outer race (OR) fault signal of Case Western Reserve University (CWRU) data: (**a**) time waveform; (**b**) FFT spectrum; (**c**) envelope spectrum.

The vibration signal of the OR fault is displayed in Figure 7a. The kurtosis of this signal was 3.5730. Figure 7b,c shows the FFT spectrum and envelope spectrum, respectively. From Figure 7a, some peaks appeared, but a certain amount of observable noise interference was visible. Several resonance frequency bands can be seen in Figure 7b, while *f_o_* and its associated harmonics were invisible. From Figure 7c, although an obvious peak emerged at the frequency of 105.5Hz corresponding to *f_o_*, the amplitudes of the harmonics associated with *f_o_* were too weak to be seen.

**Figure 8:**
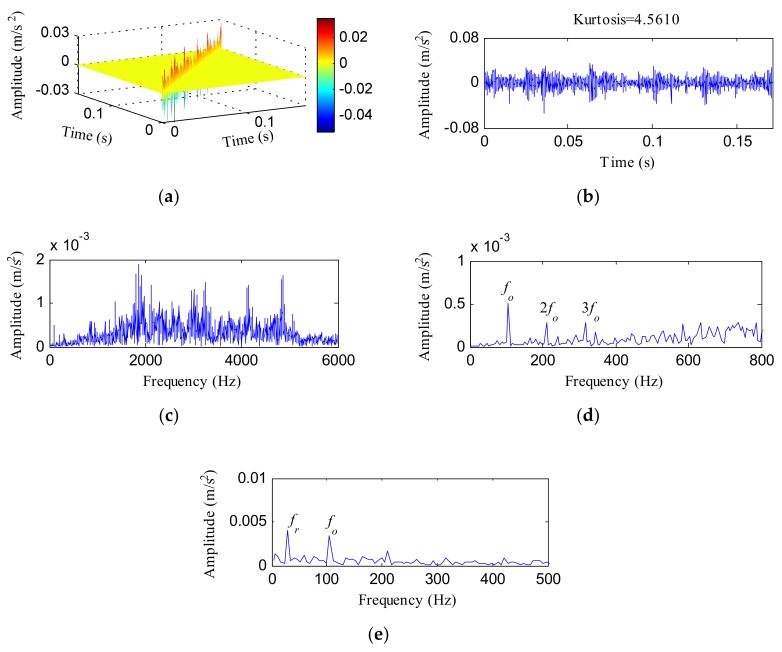
The analysis results of the OR fault signal of CWRU using the HTT transform: (**a**) the HTT transform spectrum; (**b**) the diagonal elements of (**a**); (**c**) the FFT spectrum of (**b**); (**d**) the local FFT spectrum of (**b**); (**e**) the envelope spectrum of (**b**).

**Figure 9:**
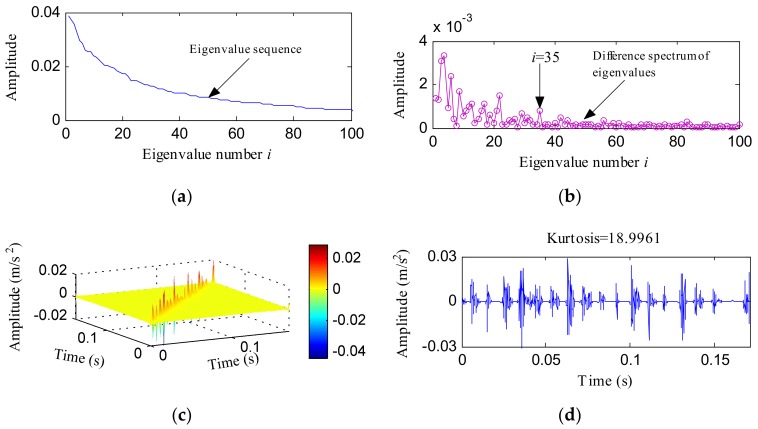

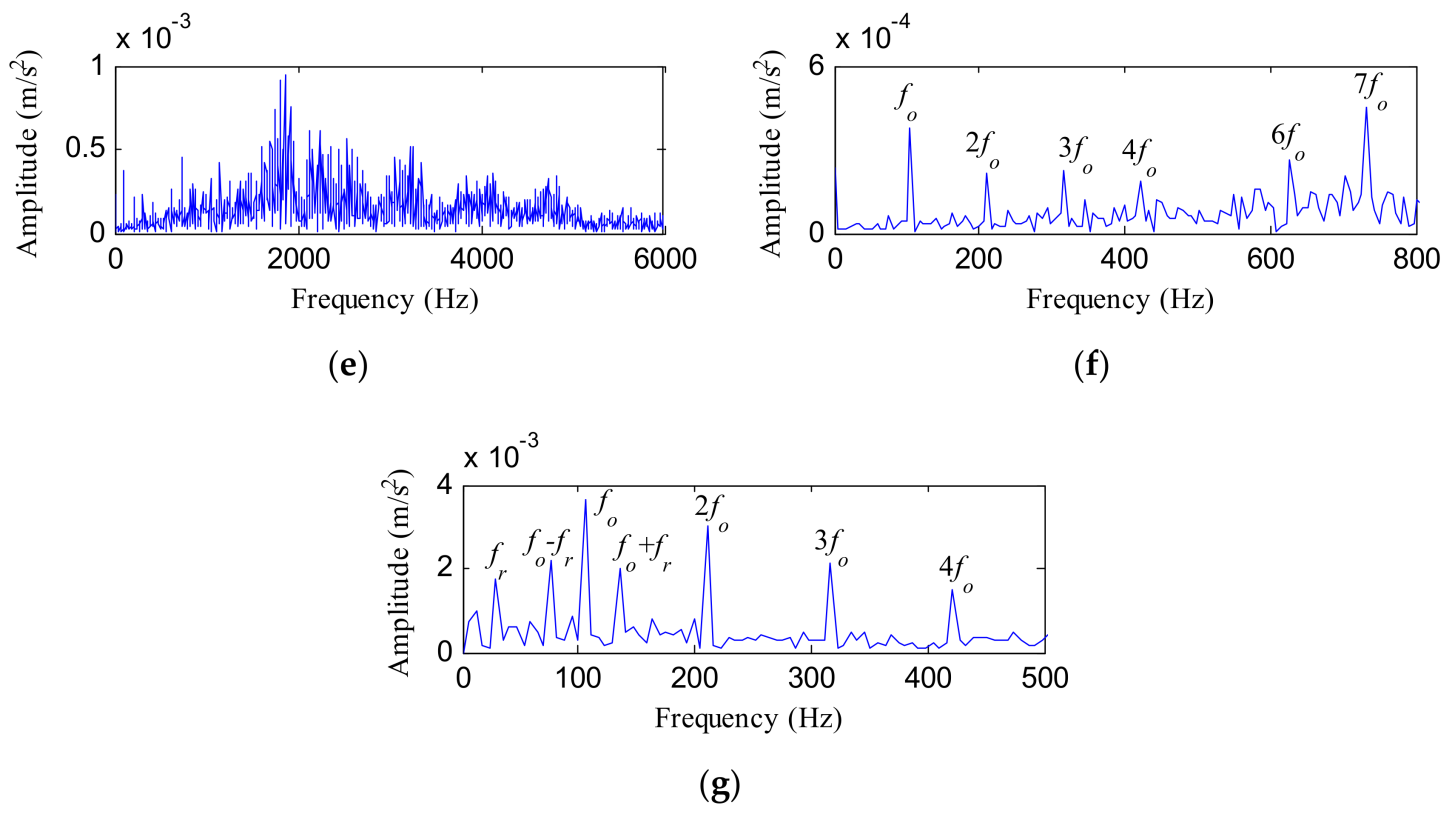
The IHTT transform process results of the OR fault signal of CWRU: (**a**) the front 100 data of the eigenvalue sequence; (**b**) the front 100 data of the difference spectrum of eigenvalues; (**c**) the IHTT transform spectrum; (**d**) the diagonal elements of (**c**); (**e**) the FFT spectrum of (**d**); (**f**) the local FFT spectrum of (**d**); (**g**) the envelope spectrum of (**d**).

The raw vibration signal was subjected to the HTT transform and IHTT transform for comparison and the results are separately depicted in Figure 8 and Figure 9. Figure 8a shows the HTT transform spectrum of the OR fault signal and Figure 8b displays its diagonal data. After the HTT transform, the kurtosis increased to 4.5610. Figure 8c shows the FFT spectrum of Figure 8b. The local spectrum of Figure 8c at the frequency range of 0–800 Hz is displayed in Figure 8d, from which we can identify three clear peaks at the frequencies of 105.5, 210.9 and 316.4 Hz, which correspond to *f_o_*, 2*f_o_* and 3*f_o_*, respectively. Figure 8e plots the envelope spectrum of Figure 8b, and two main frequencies (*f_r_* and *f_o_*) were observed in this spectrum. When applying PCA to the HTT transform matrix shown in Figure 8a, we get the corresponding eigenvalue sequence and the difference spectrum of eigenvalues, which are, respectively, displayed in Figure 9a,b. The front 35 principal components and the corresponding eigenvectors were used to reconstruct the de-noised matrix based on the difference spectrum of the eigenvalues and Figure 9c shows the obtained IHTT transform spectrum. Figure 9d shows the diagonal data of Figure 9c. The kurtosis value ascended to 18.9961. The signal also exhibited stronger impact characteristics, with the FFT result is displayed in Figure 9e. Figure 9f illustrates the local frequency band between 0 and 800 Hz of Figure 9e. From Figure 9f, *f_o_* and more of its harmonics stood out compared with Figure 8d. The envelope result of the data in Figure 9d is plotted in Figure 9g. Not only could more harmonic frequencies of *f_o_* be identified in Figure 9g compared with Figure 8e, but the modulation frequency *f_r_* and the side frequency (*f_o_* ± *f_r_*) could also be clearly detected.

**Figure 10:**
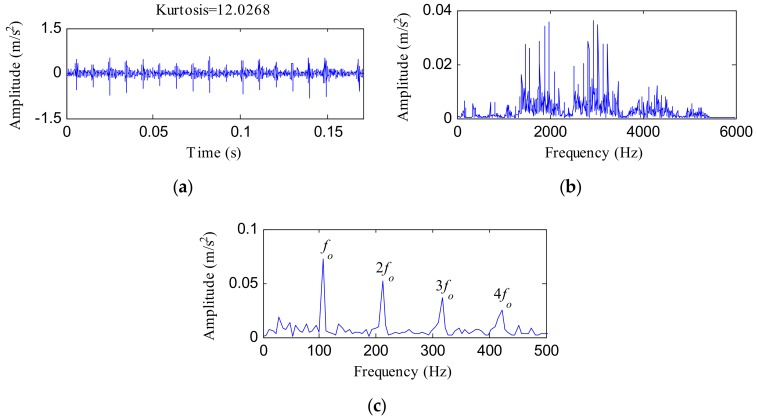
(**a**) MED filtered signal of the OR fault signal; (**b**) its FFT spectrum; (**c**) its envelope spectrum.

Figure 10 shows the analysis results using MED. The MED filtered signal is depicted in Figure 10a. The kurtosis value came to be 12.0268, which was lower than the value achieved using the IHTT method. Figure 10b,c reflects the associated FFT spectrum and envelope spectrum, respectively. No fault characteristic information was found, as shown in Figure 10b. Although significant peaks appeared at the frequencies of *f_o_*, 2*f_o_*, 3*f_o_* and 4*f_o_*, as Figure 10c shows, the MED method failed to extract the modulation information. 

### 5.2. Case 2: Inner Race Fault Detection

**Figure 11:**
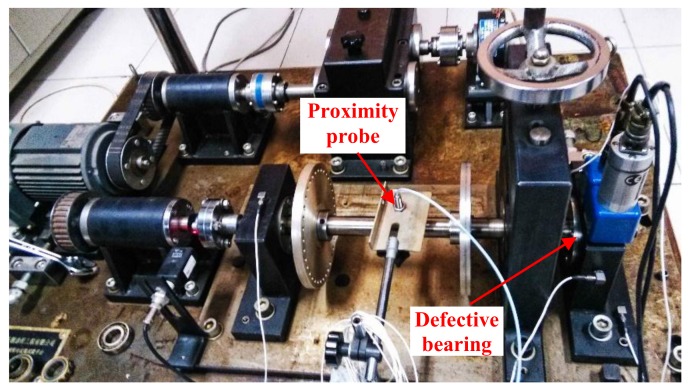
Test rig of case 2.

**Figure 12:**
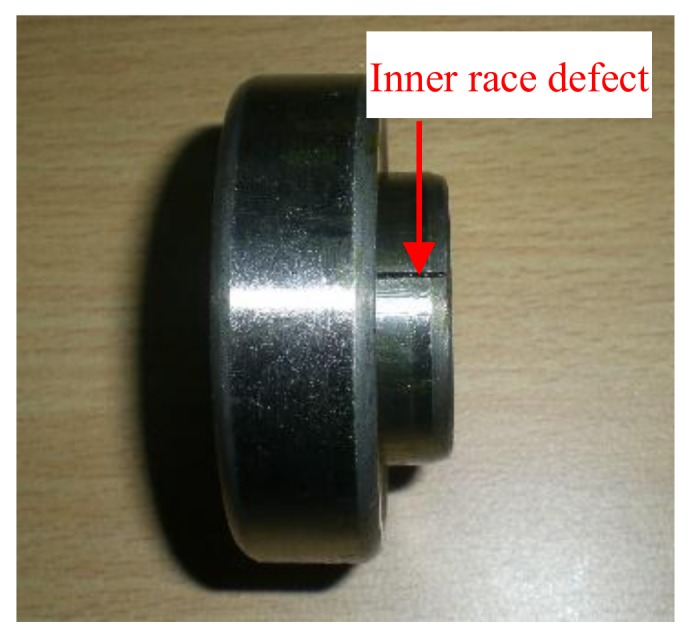
Bearing with inner race fault.

The test bench shown in Figure 11 was adopted to simulate an inner race (IR) defect and the position of the defective bearing is also shown in this figure. The type of the tested defective bearing, as Figure 12 shows, was a *N*205 cylindrical rolling bearing. Table 2 provides the specific introduction of the tested bearing. During the test, the rotating frequency of the drive shaft was set to 24 Hz, and a proximity probe was installed near the drive shaft to acquire the displacement vibration signal, with a sampling rate of 12800Hz. The IR defect characteristic frequency (*f_i_*) was calculated as 172 Hz.

**Figure 13:**
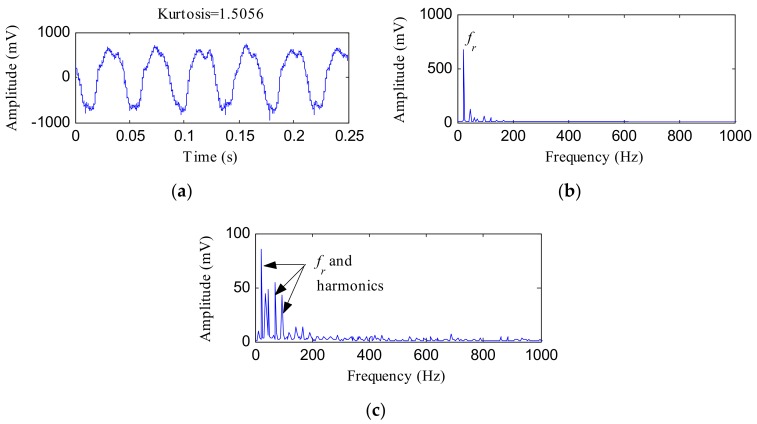
The inner race (IR) fault signal: (**a**) time waveform; (**b**) FFT spectrum; (**c**) envelope spectrum.

Figure 13 displays the time waveform, FFT and envelope spectrums of the raw displacement vibration signal. The kurtosis of this signal was 1.5056. There were no high frequency bands that might have carried the fault features in the FFT spectrum, as Figure 13b shows, and the main frequency in this spectrum was *f_r_*. In Figure 13c, *f_r_* and its harmonics could be detected. No fault features could be detected in Figure 13b,c.

**Figure 14:**
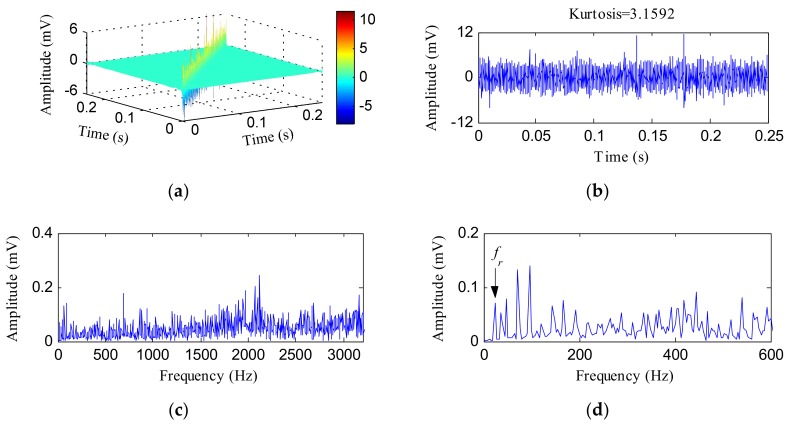

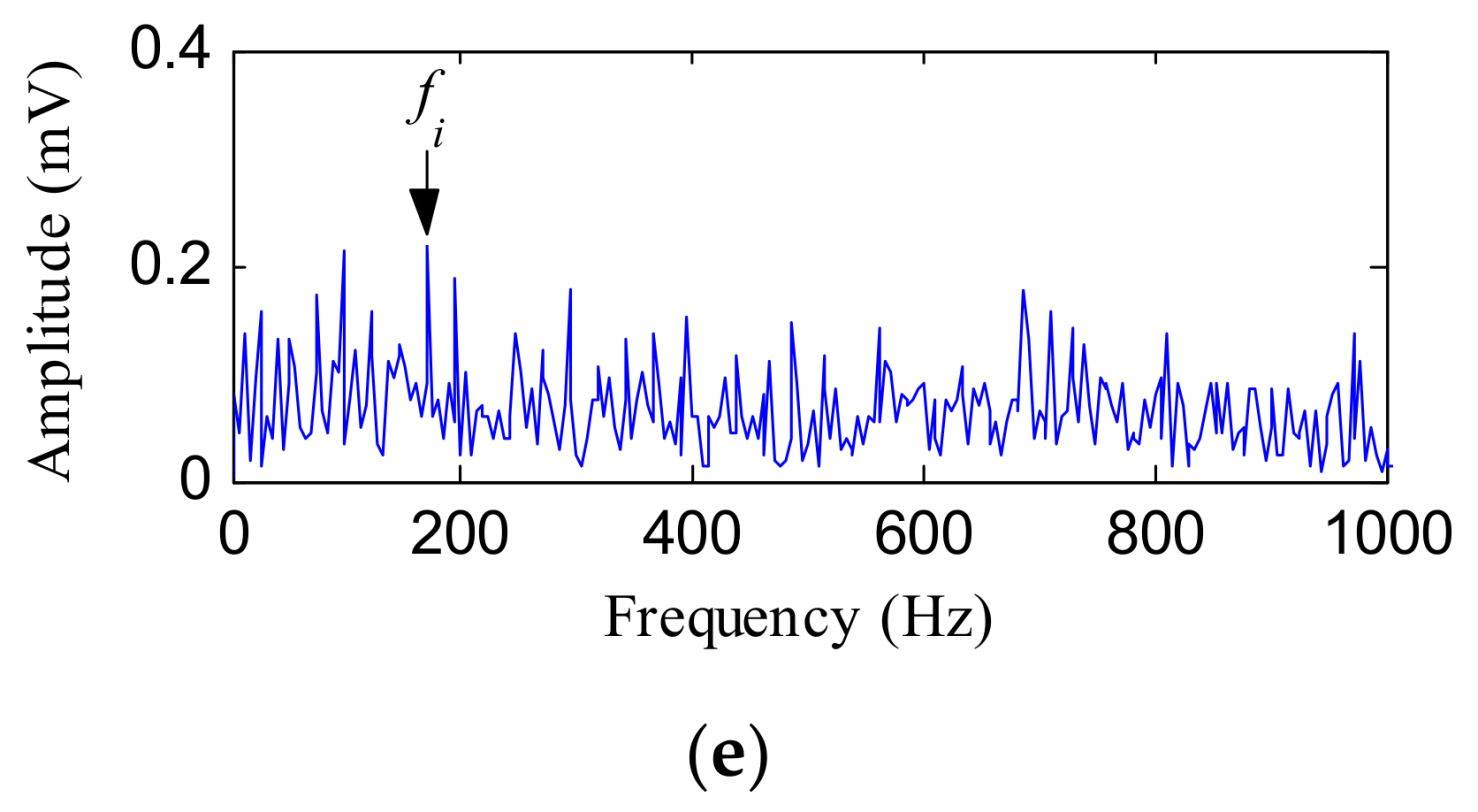
The analysis results of the IR fault signal using the HTT transform: (**a**) the HTT transform spectrum; (**b**) the diagonal elements of (**a**); (**c**) the FFT spectrum of (**b**); (**d**) the local FFT spectrum of (**b**); (**e**) the envelope spectrum of (**b**).

Figure 14 shows the HTT transform analysis results of the IR fault signal. The obtained HTT transform spectrum is shown in Figure 14a. Figure 14b shows its diagonal data. The kurtosis value increased to 3.1592 after the HTT transform. The FFT spectrum of Figure 14b is plotted in Figure 14c, and some high frequency components were visible in this spectrum. Figure 14d is the local spectrum of Figure 14c, in which we observed that some peaks corresponded to *f_r_* = 24Hz and its harmonics. The envelope spectrum of Figure 14b is displayed in Figure 14e. From Figure 14e, the fault characteristic frequency *f_i_* was identified, but many interference frequencies could also be visible.

**Figure 15:**
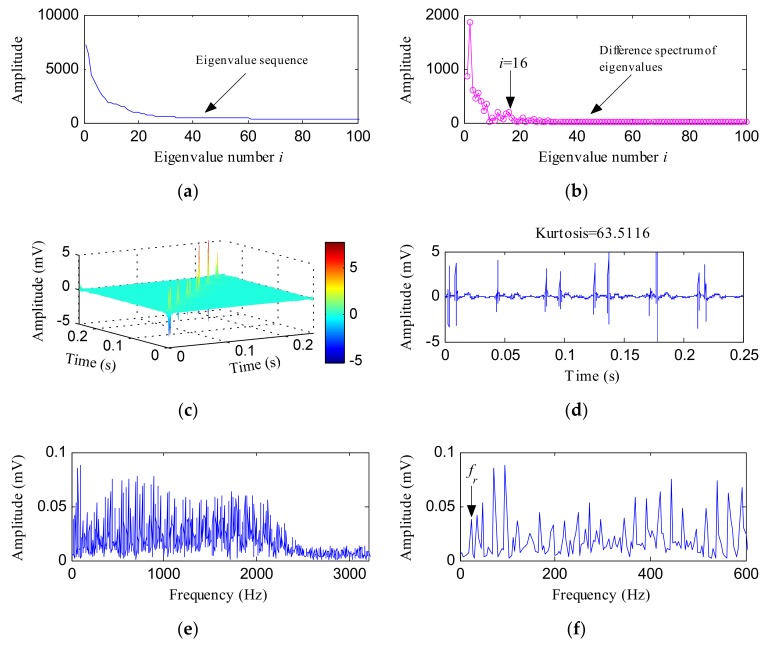

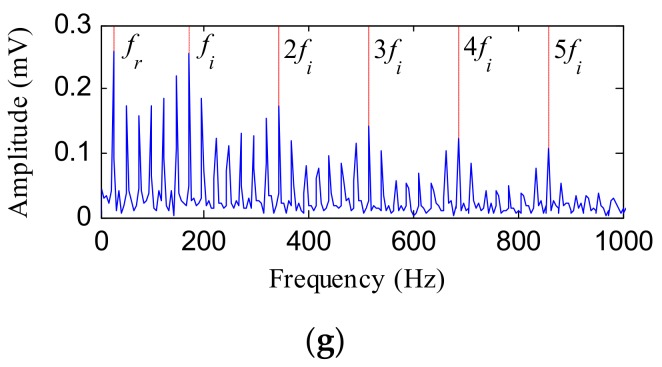
The IHTT transform process results of the IR fault signal: (**a**) the front 100 data of the eigenvalue sequence; (**b**) the front 100 data of the difference spectrum of eigenvalues; (**c**) the IHTT transform spectrum; (**d**) the diagonal elements of (**c**); (**e**) the FFT spectrum of (**d**); (**f**) the local FFT spectrum of (**d**); (**g**) the envelope spectrum of (**d**).

Furthermore, the IHTT transform was performed on the IR fault vibration signal. First, the HTT transform matrix was de-noised by the PCA method, and the obtained eigenvalue sequence and difference spectrum of eigenvalues are, respectively, shown in Figure 15a,b. The IHTT transform spectrum is shown in Figure 15c. By extracting the diagonal elements, we get the signal shown in Figure 15d. More obvious impulsive signatures could be identified in Figure 15d and the kurtosis value jumped to 63.5116. Figure 15e,f exhibits the FFT spectrum and local FFT spectrum of Figure 15d, respectively. The local FFT spectrum reflects many peaks at the frequencies of *f_r_* and its associated harmonic frequencies. From Figure 15g, the envelope spectrum of Figure 15d, we found obvious peaks at the frequencies of *f_i_*, 2*f_i_*, 3*f_i_*, 4*f_i_*, and 5*f_i_*. The sidebands were also very conspicuous which reflects the modulation between the fault characteristic frequencies and *f_r_*.

**Figure 16:**
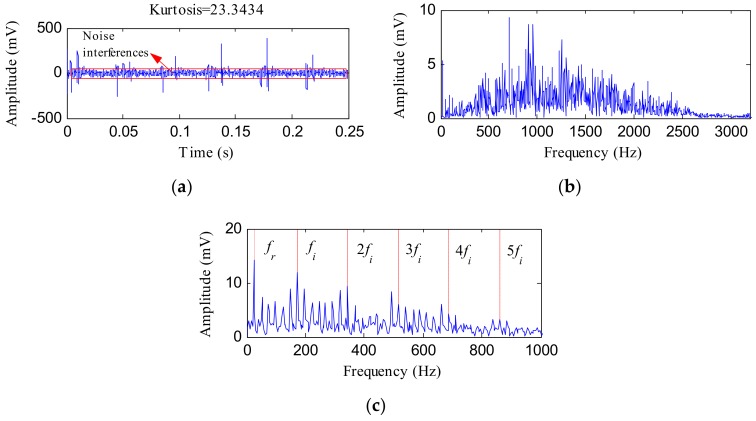
(**a**) MED filtered signal of the IR fault signal; (**b**) its FFT spectrum; (**c**) its envelope spectrum.

By employing MED to the original IR fault signal, we get the MED filtered signal displayed in Figure 16a. The kurtosis of the filtered signal came to be 23.3434, which was lower than the value obtained using the IHTT transform. Moreover, the noise interferences of Figure 16a were more noticeable than that of Figure 15d, which reflects that MED is less robust to noise compared with the presented method. Figure 16b,c displays the FFT and envelope results of the filtered signal, respectively. From Figure 16c, only the first three harmonic frequencies of *f_i_* were obvious, whereas the fourth and fifth harmonic frequencies of *f_i_* and their sidebands were less evident compared with Figure 15g. This is because the noise interferences of the filtered signal obtained using MED decrease the impact characteristics of the fault feature signal. The presented method shows advantages in inhibiting noise interferences and extracting the harmonics of IR fault characteristic frequencies.

## 6. Conclusions

An IHTT transform method was proposed in this paper to enhance and extract the weak fault features of defective bearings by combing PCA with an HTT transform. In practice, the fault impact signal generated by the defective bearing was easily submerged by noise and harmonic interferences. Benefited by the features of TT transforms, the HTT transform method inhibits harmonic interference by extracting the diagonal data of the HTT transform matrix; however, it can be still affected by noise. With the application of PCA to de-noise the HTT transform matrix, we can suppress noise significantly and get the IHTT transform spectrum. The diagonal data of the IHTT transform spectrum gives more obvious shock features and purer results. Both simulated and experimental verifications were employed to investigate the proposed method. The results demonstrated that the IHTT transform ameliorated the analysis effects of the HTT transform when analyzing noise pollution signals. The IHTT transform method therefore showed an advantage for improving impulsive fault feature signals.

## Figures and Tables

**Table 1:** The parameters of the SKF 6025 deep grove ball bearing.

Roller diameter	Pith diameter	Number of the roller	Contact Angle
7.94 mm	39 mm	9	0°

**Table 2:** The parameters of the N205 cylindrical rolling bearing.

Roller diameter	Pith diameter	Number of the roller	Contact Angle
7.5 mm	38.5 mm	12	0°
